# Two-Photon Imaging of Calcium in Virally Transfected Striate Cortical Neurons of Behaving Monkey

**DOI:** 10.1371/journal.pone.0013829

**Published:** 2010-11-04

**Authors:** Barbara Heider, Jason L. Nathanson, Ehud Y. Isacoff, Edward M. Callaway, Ralph M. Siegel

**Affiliations:** 1 Center for Molecular and Behavioral Neuroscience, Rutgers University, Newark, New Jersey, United States of America; 2 System Neurobiology Laboratories, The Salk Institute for Biological Studies, La Jolla, California, United States of America; 3 Department of Molecular and Cell Biology, University of California, Berkeley, California, United States of America; Cedars-Sinai Medical Center and University of California Los Angeles, United States of America

## Abstract

Two-photon scanning microscopy has advanced our understanding of neural signaling in non-mammalian species and mammals. Various developments are needed to perform two-photon scanning microscopy over prolonged periods in non-human primates performing a behavioral task. In striate cortex in two macaque monkeys, cortical neurons were transfected with a genetically encoded fluorescent calcium sensor, memTNXL, using AAV1 as a viral vector. By constructing an extremely rigid and stable apparatus holding both the two-photon scanning microscope and the monkey's head, single neurons were imaged at high magnification for prolonged periods with minimal motion artifacts for up to ten months. Structural images of single neurons were obtained at high magnification. Changes in calcium during visual stimulation were measured as the monkeys performed a fixation task. Overall, functional responses and orientation tuning curves were obtained in 18.8% of the 234 labeled and imaged neurons. This demonstrated that the two-photon scanning microscopy can be successfully obtained in behaving primates.

## Introduction

Two-photon scanning microscopy (TPSM) has permitted the visualization of single neurons both *in vitro* [1] and *in vivo* [2]. Neurons are labeled with fluorescent proteins, often reporters of function (e.g., calcium or voltage sensors), and the tissue penetrating infrared laser beam is used to excite and record from the labeled cells. As both morphology and function can be visualized in large assemblies of neurons, questions of simultaneous activity of neurons within a network can be addressed. For example, dynamics of spontaneous activity and spatial clustering of neuronal firing has been revealed in cortical slice preparations [3].

Perhaps the most exciting of these studies have been the application in intact animals. The high resolution of TPSM allows detailed visualization of dendritic spines and has revealed that experience alters their shape as a function of neuronal activity [4], [5], [6]. The activity of populations of neurons has been assessed as to the fine structure of the cortical architectures in anesthetized rodents and cats [7], [8]. Studies examining the activity of populations of neurons in awake animals have been restricted to small rodents – rat and mouse [9], [10], [11]. However these studies typically do not address long-term issues such as visual processing, memory or plasticity because certain fluorescent proteins and voltage sensitive dyes may cause damage in the neuronal tissue over such time scales [12], [13], [14].

Measurements in behaving monkey have long been associated with questions of neuronal activity arising from higher cognitive processing, such as visual processing [15], spatial attention [16], object recognition [17], and executive function [18] often with single unit recordings. The behaving monkey preparation was developed by Evarts and Mountcastle [19], [20]. A monkey is trained to a behavioral task for which a particular aspect of cognition can be examined. To test the correlation between cognition and neural function, one or more electrodes are inserted into the cortical area of interest via an aseptic chamber through the intact dura. The activity of single cells, and more recently local field potentials, are electrophysiologically measured while the animal performs a cognitive task. At the end of each experimental day, the electrode is removed, the aseptic chamber sealed and the animal returned to the cage. While such recordings can be repeated daily for weeks to years, the location and identity of the neurons is not known. Some studies have categorized extracellularly recorded neurons into pyramidal and interneurons based on the spike shape [21], [22], [23], [24], [25]. However, there have been questions raised whether these are truly distinguished [26]. Furthermore, although these electrophysiological experiments may leave one with a profound understanding of the neural properties, they do not provide the location and neuronal mechanism crucial for elucidating the means by which individual cells give rise to cognition. Large-scale imaging methods such as intrinsic or voltage-sensitive dye imaging repeatedly assess activity of larger neural networks with limited spatial (∼0.5–50 µm) resolution, but they fail to reveal identity or morphology of single neurons [27], [28], [29].

The present study provides a technique whereby single neurons can be recorded *in vivo* in the behaving monkey using TPSM and labeling neurons with fluorescent proteins. A genetically encoded membrane targeted calcium sensor, memTNXL, is used to measure neural activity with orientation tuning repeatedly over up to 10 months in primary visual cortex. Further studies and improvements are discussed.

## Materials and Methods

All experiments were approved by the Rutgers University Institutional Animal Care and Use Committee (Protocol #90–080) and conform to the guidelines of the National Institutes of Health. The use of non-human primates in research is outlined in the protocol and includes details of animal welfare and the steps taken to ameliorate suffering in all work involving non-human primates. All experiments were performed to ensure the animals' well-being.

### Stimulus, Task and Data Acquisition

Two monkeys (M3R, 8.5 kg and M4R, 10.5 kg) were trained to fixate a small (0.5°) red fixation point within 1°. Eye position was monitored with an infrared eye-scanner (Model RK-416, ISCAN; Cambridge, MA) at 60 Hz. A trial started with the central fixation dot, which the animal had to fixate within 400 ms. After 1500 ms, a visual stimulus consisting of a square wave grating (2.2 cycles/°, speed 0.5 cycles/s) within a circular mask (diameter 26°) appeared for 3500 ms. Contrast was 0.93 and the brightest bar was 52 cd/m^2^. At the end of the 5000 ms, stimulus and fixation point were turned off. If the monkey maintained correct fixation he received a juice reward, otherwise the trial was aborted immediately and a new trial started within 2 s. Each stimulus moved in one of eight directions, always orthogonal to the grating orientation. A blank stimulus consisting of the fixation dot only presented for the entire stimulation period was also included. Performance was at least 80% correct responses for both animals.

To visualize labeled sites and neurons, *structural* scanning was performed at higher resolution and lower scanning rate of 1 Hz (512×512 pixels). This allowed detailed visualization of the neurons within the field of view and to establish the optimal scanning depth. Subsequently, experiments continued with *functional* scanning combined with visual stimulation at higher scanning rate (4, 8, or 16 Hz) to increase temporal resolution. The higher scanning rate was achieved by decreasing vertical resolution and maintaining constant horizontal resolution (512×128, 512×64, or 512×32 pixels; 20, 40 or 80 frames per 5 s trial, respectively). One such scanning run consisted of at a minimum of 100 trials yielding about 10 trials per condition (8 motion directions and blank stimulus). The NIMH Cortex software (http://www.cortex.salk.edu) displayed the stimuli and controlled the behavioral variables (i.e., visual fixation). The start of a scanning trial was initiated by the Cortex software via a digital trigger. Scanning was performed during this entire period of 5 s to evaluate the FRET signal during baseline (fixation dot only) and stimulation (gratings or blank plus fixation dot) epochs. The stability of the imaging setup often allowed repeated scanning runs over one site.

### Rigidity of the Optical Imaging Chamber Relative to the Two-Photon Microscope

**Figure 1:**
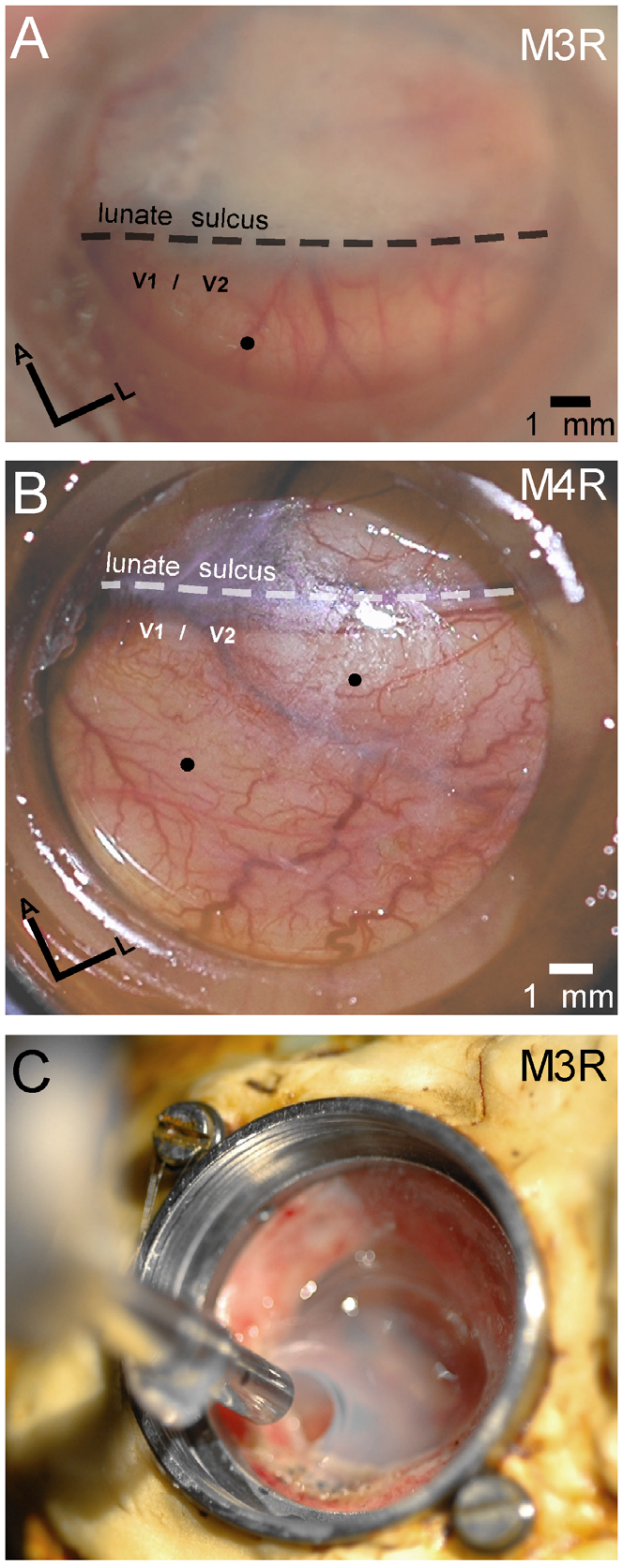
Optical chamber with artificial dura for two-photon scanning. The injections sites are indicated by black dots in V1/V2 for M3R (A) and M4R (B). Olympus Microprobe objective retracted after scanning in M3R (C). Optical chamber is filled with 2% agarose for stability.

The cap on the monkey's skull was assembled of Palacos R bone cement (Smith & Nephew, Memphis, TN) on top of 15 to 25 titanium cranio-maxillofacial screws (Synthes, Paoli, PA). Embedded in the cement cap was a head holder made of a single milled steel assembly with ¼”-20 holes to allow attachment to the supporting rail system. The optical recording chamber (20 mm inner diameter) was embedded in the Palacos cement. To allow unobstructed access to the cortex, a transparent artificial dura (M3R: 14 mm diameter; M4R: 16 mm diameter; thickness 50 µm), similar to that used previously for intrinsic optical imaging [30], [31], was implanted (Figure 1).

**Figure 2:**
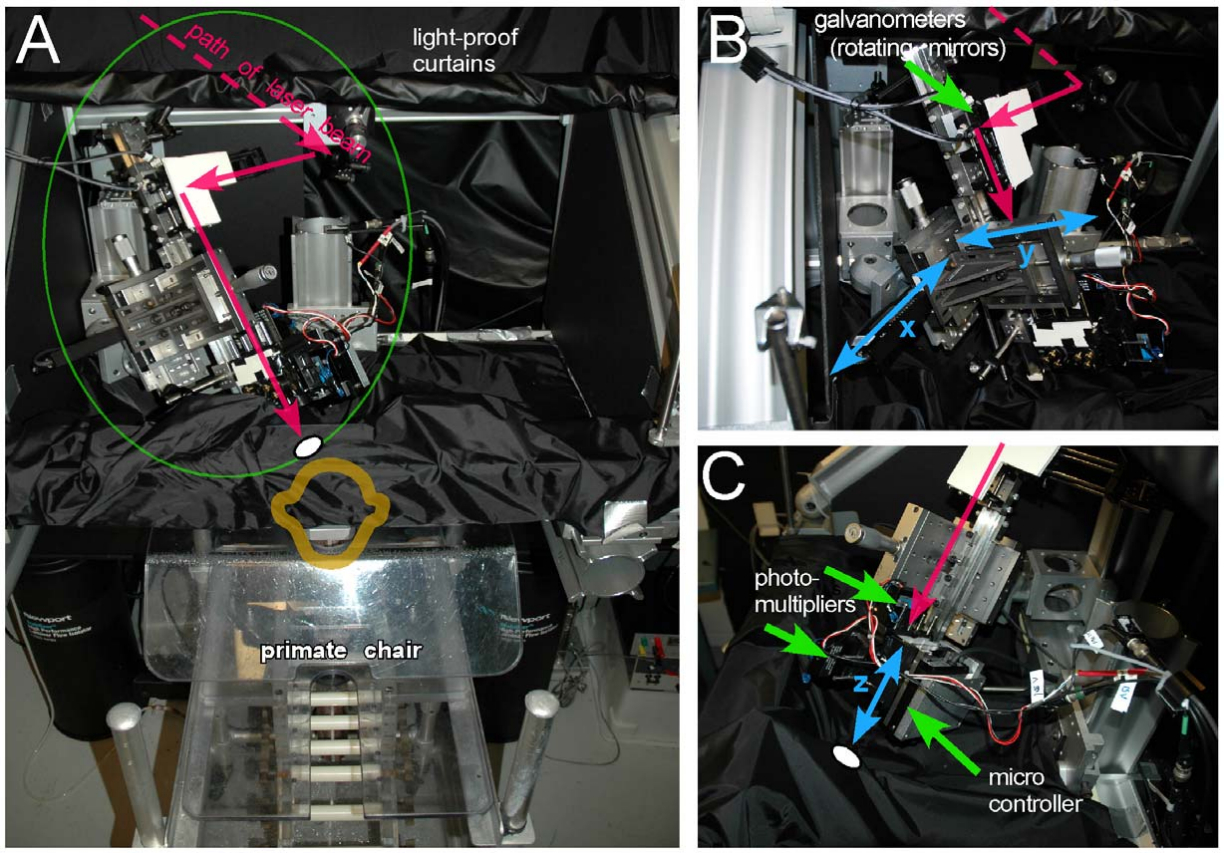
Two-photon scanning microscope (TPSM) setup for the behaving monkey. (A) Overview front of TPSM with primate chair in place. Main parts of TPSM are circled in green with magenta arrows indicating path of laser beam to be directed into monkey's chamber (white ellipse on schematic of monkey head). (B) Detail of TPSM from lateral side with motors moving medio-lateral (x-axis, blue arrow) and antero-posterior (y-axis, blue arrow). Scanning mirrors on top of microscope are indicated by green arrow. (C) Detail of TPSM from medial side with micromanipulator allowing precise vertical (z-axis, blue arrow) movement with micrometer precision.

The supporting rail system was constructed of Newport X-95 rails attached to a Newport RS2000-36–12 table on vibration isolation legs (Newport PL2000 series, Figure 2). This provided a highly rigid system for the monkey's head and chair (Crist Instrument Co., Inc., Hagerstown, MD) to be attached. This rail system also contained the TPSM. In order to achieve necessary mechanical stability of less than 1 µm movement between the two-photon microscope and the monkey's head, additional stabilizing elements were added between the rail system and the monkey's head compared to earlier intrinsic imaging experiments [30], [32].

### Two-Photon Scanning Microscope

The TPSM (Figure 2) was custom built according to published physical designs [33]. A Mai Tai Ti:sapphire Laser (Spectra-Physics, Mountain View, CA) tunable from 700 to 1020 nm projected a beam into a series of mirrors and lenses to reshape it. A half-wave plate adjusted the power of the beam. The settings used in all experiments ranged from 20–40% transmitted light, which reduced the 1800 mW at 810 nm to 360–720 mW. We saw no evidence of photo-damage from the half-wave plate adjustment of the beam power. The beam was further focused onto a pair of high-speed galvanometers (Model 6210, Cambridge Technology, Watertown, MA). The scanning beam passed through a scan lens (Thorlabs, Newton, NJ), a tube lens (Carl Zeiss, Jena, Germany), a long-pass dichroic beamsplitter (720DCXXR; Chroma Technology Corporation, Rockingham, VT) and a microscope objective, and finally reached the cortex. After excitation of the fluorophores, the emission (all below 720 nm) was reflected by a bandpass dichroic filter (510dclp, Chroma Technology, Rockingham, VT) to the two photomultiplier (PMT) modules (H7710-13; Hamamatsu, Bridgewater, NJ), each equipped with a bandpass filter (both Chroma) for detecting the emissions from the two fluorescent proteins: an HQ480/40 M for the cyan fluorescent protein (CFP), and an HQ535/40 M for the yellow fluorescent protein (YFP, citrine). The laser was tuned to 810 nm for excitation of the CFP [34]. Two low-noise current preamplifiers (SR570; Stanford Research Systems, Sunnyvale, CA) provide a signal from the PMT to the high-speed A/D converters (NI DAQCard 6036E; National Instruments, Austin, TX) in a Microsoft XP data acquisition computer (Dell Precision 650) running the ScanImage software [35].

The two-photon scanning microscope for use with the behaving monkey needed to fulfill two additional physical requirements compared to an equivalent setup for rodent or slice imaging. First, it needed to be adjustable to a range of angles and positions so that it could align with the chamber on the monkey's head. Second, a small lens was required to reach inside the chamber and be close to the cortex. The first issue was solved using a combination of mirrors and Newport parts that permitted the microscope to be placed at any position (Figure 2A). The location of this portion of the assembly was fixed relative to the monkey's head. A manual manipulator (UMR 12.40 single axis stage; Newport, Irvine, CA) attached to the stainless steel rail allowed large vertical movements. Movements in the horizontal axis were accomplished by a motorized stage (850G Linear Actuator; Newport, Irvine, CA; Figure 2B). The microscope lens was attached to the end of the cavity and could be moved up and down for a distance of approximately 150 mm along with the dichroics, filters and photomultiplier tubes upon the Newport micro-manipulator (VP-25X Precision Compact Linear stage; Newport, Irvine, CA; Figure 2C). Additional steel rods were attached to the microscope assembly for further rigidity once a site of labeled neurons was located, and only vertical microscopic movements were necessary.

The second technical obstacle was to find a microscope objective to fit into the recording chamber. Regular, high numerical aperture, water-immersion microscope lenses have a diameter of 20–40 mm with a working distance of approximately 1–3 mm (e.g., Olympus, XLUMPlanFl, 20x, N.A. 0.95, W.D. 2 mm). The implanted metal recording chamber measures 20 mm in diameter and is about 10–12 mm deep (Figure 1). Clearly, the tip of a regular lens cannot reach the surface of the artificial dura. Even a larger optical chamber with a diameter of 25 mm, as used in a previous intrinsic imaging study [29] would be too narrow for a regular microscope lens. Initially, *g*radient refractive index GRIN lens were tested, which are small enough, but these had poor optics, low numerical aperture and a small field of view [36], [37]. Long-working distance lenses (W.D. >12 mm; Optem Thales, Fairview, NY) were also tested but their optical qualities were not sufficient for resolving single labeled cells.

The only lens that met the requirements was an Olympus Microprobe objective (27X, 0.7 NA, W.D. 200 µm; Olympus, Center Valley, PA), measuring 3.5 mm in diameter and 25 mm in length. This particular design permitted access to all locations within the chamber. For two-photon imaging the lens was lowered into the open chamber under visual guidance, injection sites were located by utilizing vessel landmarks relative to the position of the Microprobe tip. Once labeled neurons were located with scanning, the chamber was filled with sterile 2% agarose in 0.9% saline (Figure 1C), and the microscope further secured to the rack to improve stability during scanning.

The Microprobe lens has a short working distance of 200 µm. Although it seemed to be a disadvantage at first, it turned out to be an advantage. Because the lens had to lean against the artificial dura, it stabilized the tissue. Once the agar was added to fill the chamber, movement artifacts from pulsations were almost completely eliminated. In order to get deeper measurements, the tissue could be compressed gently yielding a maximal depth of 300 µm corresponding to top of layer 2/3.

To establish a complete light seal of the highly sensitive PMTs from the display monitor, the entire TPSM was enclosed with light blocking materials (Blackout Material BK5 and TB4; Thorlabs, Newton, New Jersey; Figure 2A). A circular opening of 22 mm (i.e., outside diameter of optical chamber) was cut out at the level of the monkey's head. A custom made plastic holder allowed the blackout material to be wrapped tightly around the monkey's chamber. The seal for the entire TPSM system was tested prior to each experiment with the laser turned off. With complete light seal, the onset and offset of the stimulus on the display monitor was undetectable.

### Sensors

The genetically encoded calcium sensor memTNXL (AAV1-CD8-TNXL-SH; titer 1.3×10^11^ particles/ml) was injected into V1/V2 (i.e., within approximately 1–3 mm behind the lunate sulcus) in the two monkeys. This sensor contains two main components. The first component is the TNXL portion. It consists of two fluorescent proteins: a yellow fluorescent protein (YFP) and cyano fluorescent protein (CFP). For the memTNXL sensor, the YFP variant *citrine* was used, which is more resistant to photobleaching [38] than the YFP. (Throughout the term “YFP” will be used for simplicity.) The two fluorescent proteins are connected via the calcium binding moiety troponin C [39]. The sensor measures neural activity based on the fluorescence energy transfer (FRET) effect [40], [41], [42], [43], [44]. With neural activity and an influx in Ca^2+^, the conformation of the sensor changes and the fluorescent proteins come in closer proximity, thus allowing an energy transfer from the *donor* CFP to the *acceptor* YFP. The ratio between CFP and YFP fluorescence gives a linear response of FRET efficiency over calcium concentration and provides a high stability to physiological range of pH changes [39]. In transgenic flies and cultured neurons, the FRET calcium signal with TNXL has a rise time of between 430 and 590 ms and takes about one second to reach plateau [39], [40], [45], [46]. The second component of the sensor was the membrane bound protein CD8 that is a transmembrane glycoprotein on a co-receptor for the T cell [47]. The calcium sensors bind to the cell membrane via the CD8; this likely makes the memTNXL sensor faster, as the calcium changes are first detected close to the neural membrane. The third component is a PDZ interaction domain of the Shaker K+ channel at the C terminus that has no effect in the present study.

The sensor is encoded in the recombinant adeno-associated virus serotype 1 (AAV1) with a cyto-megalo (CMV) protein promoter. It should be noted that this combination served to only infect neurons [48], [49], [50], [51], [52]. By using the AAV1 vector, neurons are not compromised and expression of the sensor continues permanently [6].

The first animal (M3R) received an injection of AAV1-CMV-memTNXL at a medial location (Figure 1A). The second animal (M4R) had two injections with AAV1-CMV-memTNXL spaced ∼5 mm apart (Figure 1B). Virus injections were made through the artificial dura under aseptic conditions while monkeys were sedated with ketamine (3–5 mg/kg) in the primate chair with their heads restrained. The primary visual cortex (V1/V2) was located a few millimeters posterior of the lunate sulcus, and the 10 µm glass pipette was lowered through the artificial dura into the cortex using a Micro Manipulator (Model 1760, David Kopf Instruments, Tujunga, CA). Aliquots of high-titer virus (∼2 µl) were pressure injected at multiple locations into superficial layers using a Picospritzer II (General Valve Corporation, Fairfield, NJ) and pulled glass pipettes (inner diameter 0.94 mm; World Precision Instruments, Sarasota, FL). The sharpened pipette was first lowered 2 mm below the cortical surface and then pulled up at defined depths, and 4 injections about 0.5 µl in volume administered within a single site. Vessel landmarks around the injection sites aided in subsequently guiding the lens positioning prior to scanning.

Expression of the sensor in the transfected neurons is expected approximately 2–3 weeks after virus injection [6], [53], [54]. In the first monkey M3R, labeled neurons were imaged between 21 and 312 days post injection. In the second monkey M4R, labeled neurons were imaged between 35 and 51 days after the first injection, and again between 9 and 29 days after the second injection.

At the time this study was performed, AAV1-CD8-TNXL-SH was shown to provide a strong and highly reproducible fluorescence signal. Another variant of the troponin C-based calcium sensors (i.e., TNXXL) showed superior sensitivity, linearity, and produced changes in fluorescence even at low firing rates [46]. However, the current study was initiated prior to this publication and we were thus unable to use TNXXL. The memTNXL, although insensitive to the firing of 1–10 action potentials, was used in the current primate study [39].

As the two monkeys are highly trained in a wide range of tasks other than that used here, e.g. attentional tasks [31], [55], their brains are not available for *post-mortem* tissue.

### Data Analysis

The CFP and YFP signals were measured separately by the two photomultipliers, analog-to-digital converted and stored in TIF files using the ScanImage software [35]. For analysis they were first dark current corrected, and then the corrected CFP and YFP data stored in Khoros format (Khoral Inc., Albuquerque, NM). Incorrect trials (e.g., break in fixation) or trials with excessive movement artifacts were excluded from further analyses.

Regions of interest (ROI) were selected by first averaging the YFP images across all good trials and rectangular regions containing neurons were delimited by eye. The mean brightness of each ROI was extracted, and the ratio of the CFP and YFP signals calculated. This YFP/CFP ratio was computed separately for the baseline duration (500 ms prior to stimulus onset) and for the evoked signal after stimulus onset. For the orientation tuning, the evoked response was taken 500–2500 ms after stimulus onset and divided by the baseline signal. This baseline normalized response was analyzed with a linear regression with the horizontal (*a_x_*) and vertical (*a_y_*) component of the grating orientation. Regression coefficients were computed for either the directional response (8 directions) or combined for the orientation response (4 orientations). Neurons were classified according to whether the directional or orientation regression coefficients reached the level of significance. The level of significance was at least P<0.05 taken from the procedure GLM of the SAS package [56].

## Results

In order to record from intact neurons in the behaving monkey with a calcium sensor, two steps have to be achieved. First, the gene product (i.e., expression of the fluorescent proteins) and long-term stability (e.g., absence of photodamage) have to be confirmed in live monkey neurons. Second, the calcium sensor has to demonstrate activity that commensurates with neural firing.

### Morphology of memTNXL Expression in Monkey Striate Cortex

In order to perform the optical studies repeatedly in time, either the natural dura would have to be opened on a regular basis or a transparent artificial dura needed to be inserted. The first route turned out not to be feasible because the underlying tissue would be traumatized. In a series of four studies, the artificial dura allowed repeated intrinsic imaging experiments up to 5 years in the same animal [29], [30], [31], [32]. Thus, the same preparation ensured that the two-photon imaging should be stable through the artificial dura and that multiple recordings could be made.

At the beginning of each recording session, the two-photon microscope with the Microprobe lens was lowered into the chamber under visual guidance until the tip just touched the artificial dura. The depth of the surface of the artificial dura and the brain was noted by reflections and increases in background fluorescence, respectively. Subsequently, the microscope was lowered with micrometer steps until labeled structures became visible.

**Figure 3:**
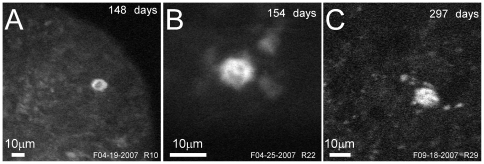
Expression of memTNXL in monkey visual cortex over time. Various time points show different sites with expression of the fluorescent sensor. (A) 148 days post injection (190 µm depth). (B) 154 days post injection (300 µm depth). (C) 297 days post injection (110 µm depth). Labeled neurons vary in size (from 10 to 20 µm). (A–C) YFP images averaged over 10 frames at 512×512 pixels resolution.

About four months after the injection of memTNXL in M3R, clearly labeled cells were imaged (Figure 3A and 3B). In these two examples, the expression level was quite high so that labeled cells were clearly discernible against the background. The structural image (Figure 3A) also shows the border of the Microprobe's circular field of view. Approximately 10 months post injection, the single labeled neuron was imaged again (Figure 3C). At this later time, it became more difficult to see fine details, presumably because of the growing tissue underneath the artificial dura. With averaging, neurons could be selected and imaged at higher resolution.

**Figure 4:**
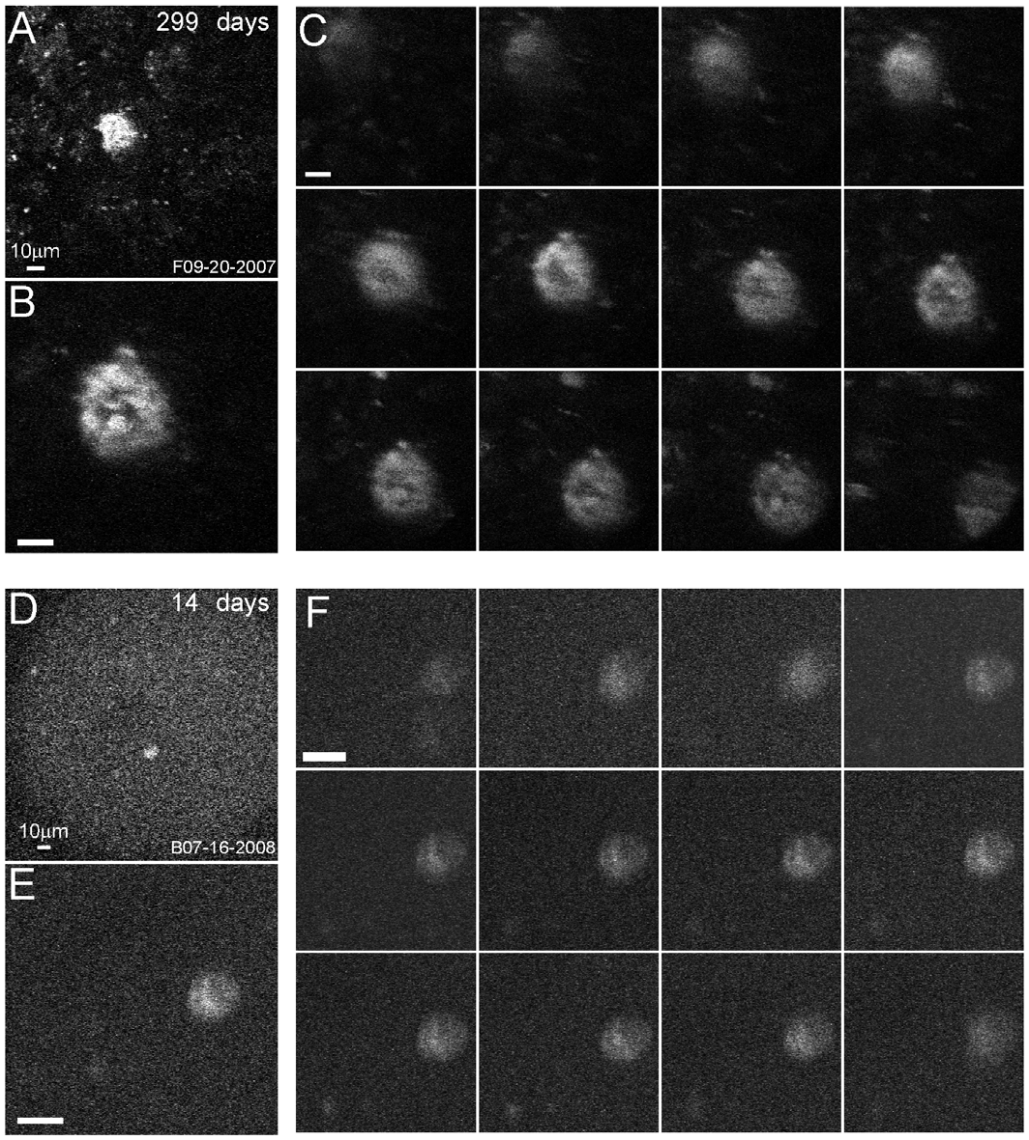
Z-stacks of two single neurons. (A) Low magnification structural overview (152×152 µm field, M3R, 200 µm depth). (B) Higher magnification image (75×75 µm). (C) Structural images collected at 12 different depths (2 µm depth intervals). (D) Low magnification structural overview (200×200 µm field, M4R, 240 µm depth). (E) Higher magnification image (60×60 µm). (F) Same as Figure 4C. (A–F) YFP images averaged over 10 frames at 512×512 pixels resolution.

To illustrate the depth dimension, vertical scans (z-stacks) were collected in both animals with sections of 2 µm intervals (Figure 4). The YFP images of the first example cell (Figure 4A and 4B) indicate that the memTNXL was located in a punctate manner near the cell membrane as expected from the inclusion of the CD8 marker. Similar expression but somewhat fainter was found in the second example (Figure 4D and 4E). The vertical scans reveal the pattern of labeling in both animals with concentration of the fluorescent protein in the cell membrane (Figure 4C and 4F).

**Figure 5:**
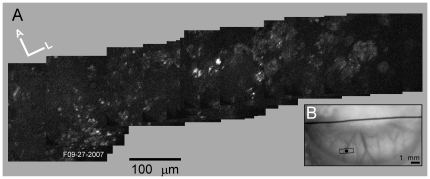
Horizontal scan along medio-lateral axis across the memTNXL injection site (M3R). (A) Montage of TPSM images with brightly labeled neurons approximately in center. Each YFP image is an average of 10 frames at 512×512 pixels resolution. (B) Location of TPSM scanning sites for montage (small rectangle over medial injection site) on photograph of brain surface.

Expression of memTNXL was not strictly confined to the injection sites but could be seen up to several hundred µm laterally away from the injection sites. At ten months post injection, a montage of YFP images was created showing labeled cells and surrounding neuropil (Figure 5A). For this montage, the microscope was moved along the medio-lateral axis and images obtained approximately every 100 µm (Figure 5B). Label up a few hundred µm from the injection site might be due to anterograde and/or retrograde transport of the AAV1 virus and the genetic material over the time period of months [57], [58], [59]. Alternatively, there may have been some spread of the virus during the injection procedure, although it would have expected to be a small amount based on the short life of the virus itself (1–2 days) and the expected dilution of the viral particles.

Repeated experiments revealed that labeling was visible over many months provided that the injection site remained optically accessible. In the first animal (M3R) scanning was performed successfully over a period of 10 months. In the second animal (M4R) the cortex was accessible for 3 months post-injection. (In both animals, regrowth of dural tissue under the artificial dura precluded scanning after 10 and 3 months.)

### Recordings in Striate Cortex During Behavior

Having recorded from morphological single neurons in behaving monkey by means of structural scanning, the functional selectivity of the calcium sensor memTNXL embedded in living neurons in V1/V2 was examined by presenting oriented gratings. Such grating stimuli are well known to yield single unit orientation selective responses in superficial neurons of the primary visual cortex in behaving monkeys [60], [61], [62], [63].

The FRET signal was assessed in a total of 234 single neurons (185 in M3R and 49 in M4R. One functional scanning run was performed at least once over each cell. For some cells, repeated functional scans were performed resulting in a total of 480 total functional scans. The majority of labeled sites contained multiple neurons, which allowed assessing responses of up to 6 neurons simultaneously. Labeled cells responding to visual stimulation are expected to show an increase in the FRET signal compared to the baseline signal when stimulated optimally.

Examples of significant cells are shown with neurons numbered consecutively throughout. Typical directionally tuned responses from three example neurons (*Neuron 1* and *Neuron 2* from M3R, *Neuron 3* from M4R) illustrate single labeled cells that were scanned at high magnification, each with a significant modulation, as confirmed by the regression coefficients.

**Figure 6:**
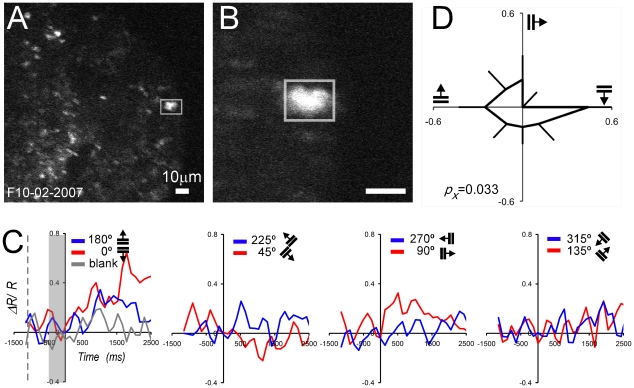
Functional TPSM of single *Neuron 1* (M3R, 311 days post injection, 260 µm depth). (A) Low magnification structural image (200×200 µm) with ROI (white rectangle) submitted to further analysis. (B) High magnification structural image (50×50 µm) with brightly labeled neuron in center. (A,B) YFP images (10 frames averaged). (C) FRET signal plotted as a function of time for all 8 directions (blue and red curves). First panel also shows blank signal (gray curve), onset of fixation (dashed line), and 500 ms baseline signal (gray shaded rectangle) used for normalization (stimulus onset was at 0 ms). (D) Circular tuning curve (mean and standard error).

*Neuron 1* was isolated by means of structural scanning at low (Figure 6A) and high magnification (Figure 6B) and appeared as a bright illuminated “blob” (∼10 µm) on a dark background taken at 512×512 pixels resolution. The functional measurements were performed over a region encompassing the labeled cell (white rectangle). For this particular neuron, a region of 50×50 µm was scanned at 512×64 pixels resolution. The vertical scanning resolution was decreased by a factor of 8 to increase the acquisition rate to 8 Hz. The cell's baseline normalized responses were plotted over time (−1500 to 2500 ms, Figure 6C). The downward moving horizontal gratings (0°, red line) elicited an FRET response of about 0.45 ΔR/R. The upward horizontal gratings (180°, blue line) increased the FRET signal slightly less at about 0.25 ΔR/R. The non-preferred directions and the blank stimulus (gray line) modulated the cell only minimally (0.1 to 0.15 ΔR/R). Plotting the means and standard errors for all directions (Figure 6D) illustrates the significant orientation tuning of the neuron (*p_x_* = 0.033).

**Figure 7:**
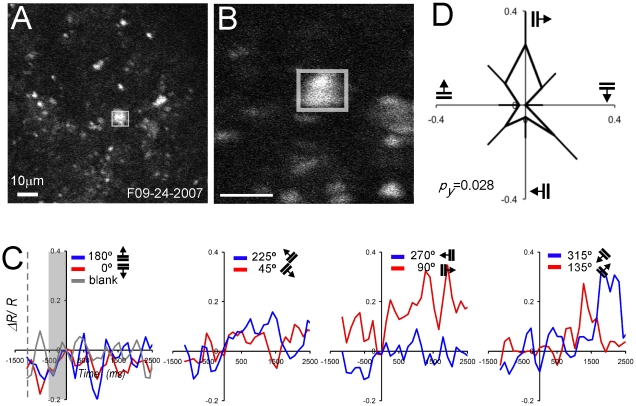
Functional TPSM of single *Neuron 2* (M3R, 303 days post injection, 150 µm depth). (A) Low magnification structural image (100×100 µm) with the ROI (white rectangle). (B) High magnification structural image (38×38 µm) with brightly labeled neuron in center. (A,B) YFP structural images (10 frames averaged). (C) FRET signal plotted as a function of time for all 8 directions (blue and red curves). Conventions otherwise as Figure 6A and 6C. (D) Circular tuning curve (mean and standard error).

*Neuron 2* was recorded among an array of labeled cells (Figure 7A and 7B). It responded predominantly to vertical gratings moving rightward (90°, 0.25 ΔR/R) but marginally to 270° gratings (0.05 ΔR/R; Figure 7C). The orientation tuning plotted as a circular tuning curve (Figure 7D) was confirmed statistically (*p_y_* = 0.028).

**Figure 8:**
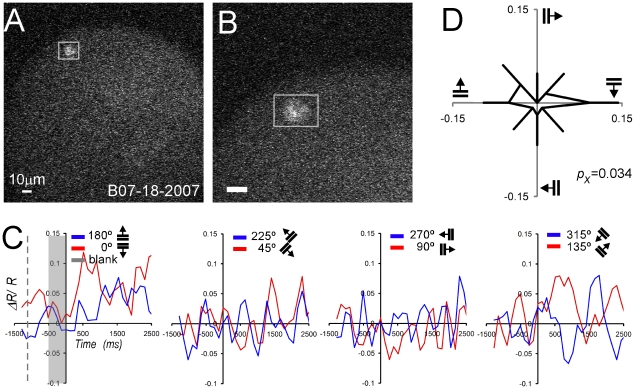
Functional TPSM of single *Neuron 3* (M4R, 14 days post injection, 300 µm depth). (A) Low magnification structural image (200×200 µm) with the ROI (white rectangle). (B) High magnification structural image (100×100 µm) with labeled neuron in center. (A,B) YFP structural images (10 frames averaged). (C) FRET signal plotted as a function of time for all 8 directions (blue and red curves). Conventions otherwise as Figure 6A–6C. (D) Circular tuning curve (mean and standard error).

*Neuron 3* was less brightly labeled than the previous two examples (white rectangle, Figure 8A and 8B) but also shows the accumulation of the fluorescent sensor in the membrane (compare with Figure 4D–4F). In this experiment, recordings started two weeks after injection. This particular neuron was tuned for horizontal gratings with an average FRET signal of 0.08 ΔR/R for the 0° direction and 0.05 ΔR/R for the 180° direction (Figure 8C). The circular tuning curve illustrates the significant orientation tuning (*p_x_* = 0.034; Figure 8D).

**Figure 9:**
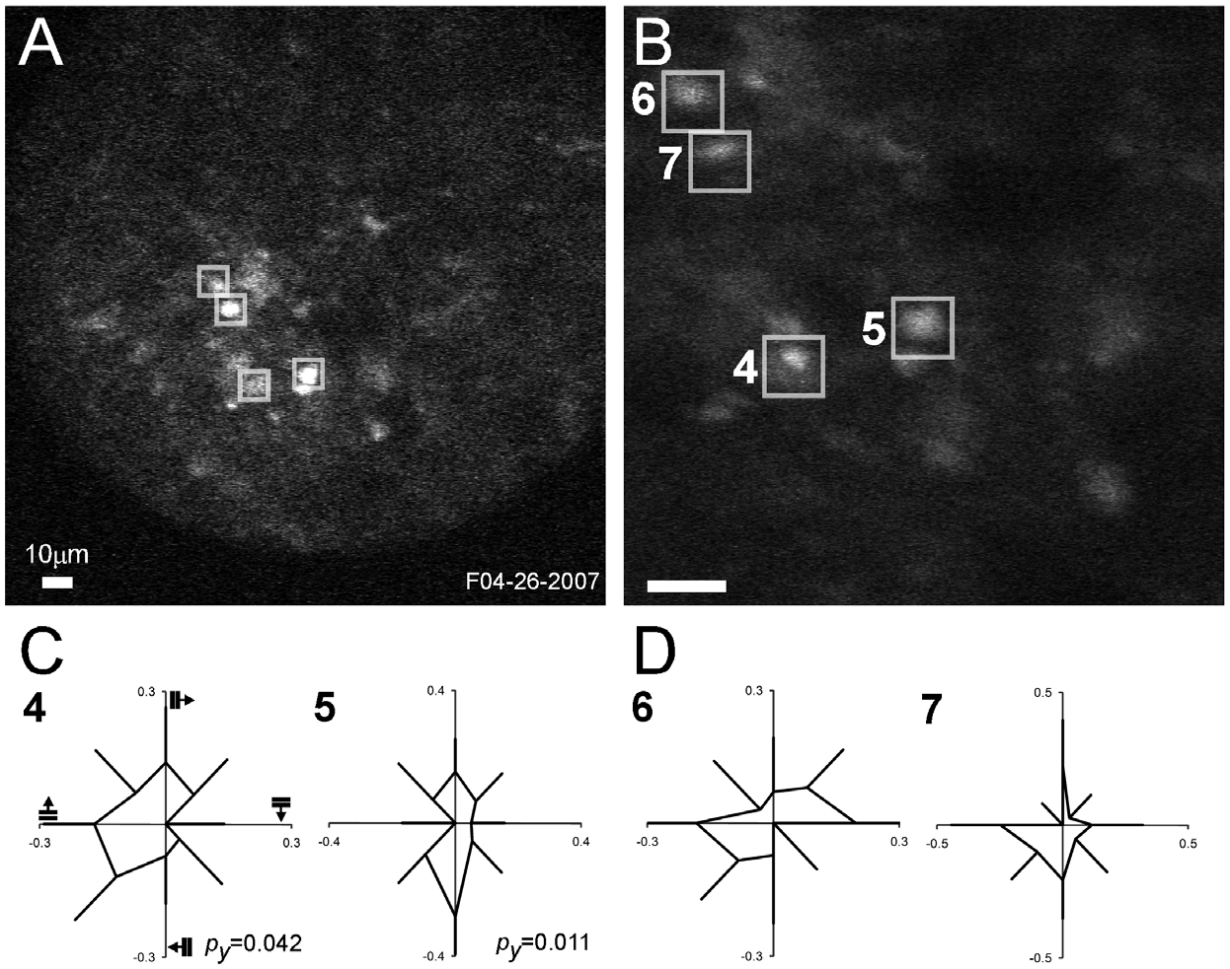
Directional responses of four selected neurons collected simultaneously (*Neurons 4–7*, 250 µm depth). (A) Low magnification structural image (200×200 µm) with the ROIs (white rectangles). (B) High magnification structural image (75×75 µm) of the same site. (A,B) YFP structural images (10 frames averaged). (C) Circular tuning curves (mean and standard error) for the two significant neurons. (D) Circular tuning curves (mean and standard error) for the two non-significant neurons.

In many experiments, recordings were made from several neurons simultaneously at lower magnification (Figure 9). This provides the possibility of determining potential relationships between different neurons during stimulation. A low magnification structural YFP image illustrates a site with brightly labeled cells (Figure 9A) of which four were selected for functional measurements (white rectangles). A high magnification image of the same site (Figure 9B) shows two neurons with significant tuning (*Neurons 4* and *5*). Two additional neurons about 20–30 µm away were also scanned but showed non-significant tuning (*Neurons 6* and *7*). The circular tuning curves are plotted for the significant (Figure 9C) and non-significant (Figure 9D) cells. *Neuron 4* responded broadly to gratings of between 225° to 45° and was significantly orientation tuned (*p_y_* = 0.042). *Neuron 5* responded predominately to vertical gratings moving left resulting in a significant directional tuning (*p_y_* = 0.011). Although *Neurons 6* and *7* were not significantly tuned, their directional preferences were similar to those of the two significant cells. The distribution of directions suggests that all arise from a neighboring cluster of orientation tuned neurons. The two distant cells might also be out of plane so that their responses were not significant.

### Population analysis

Counting the cells with significant regression parameters yielded a total of 18.8% (44/234) of recorded cells that were significantly altered by the orientation or direction of the stimulus. The preferred orientations from all significant neurons were subjected to a Rayleigh test of uniformity. This test confirmed that the preferred orientations of all 44 cells were distributed uniformly (*p* = 0.63).

## Discussion

The current study demonstrates the usage of TPSM in primary visual cortex of behaving primates. The viral transformation with AAV1 permitted the expression of the calcium sensor memTNXL and allowed the visualization of transfected neurons. These neurons were seen as bright fluorescent spots of various sizes (∼10–20 µm) against the background of the darker undifferentiated and unlabeled tissue. When tested for selectivity to gratings, the neurons responded by an increase in the FRET signal ranging at times up to 5% of the pre-stimulus level. A total of 18.8% of the 234 neurons tested showed significant directional or orientation tuning. In coupling visual stimulation in behaving monkey with TPSM, we demonstrate a striking new way to image neurons and measure their activity repeatedly over days. These advancements facilitate studies of neuron interaction and plasticity in primate species. Successful morphological *and* functional TPSM in awake behaving monkey was achieved with the following technical developments.

### Sensor and Viral Transfection

Long term fluorescent labeling of neurons with a FRET based calcium sensor memTNXL was achieved by transfecting neurons with the viral vector AAV1. This virus has low cytotoxicity and high transfection rate [6], [50], [52]. Transfection rate *in vivo* in primate cortex is not precisely known. However, in rodent cortical cultures, transfection rate with AAV1 is about 90% of all cells that are transfected of which 80% are neurons [51]. However, due to differences between species and preparation, transfection rates in primate cortex may vary.

The TNXL calcium reporter of memTNXL is expected to be targeted to the plasma membrane of the transfected cells by the integral membrane protein CD8 to which it is fused [47]. The punctate appearance of the sensor in primate cortex suggests that some of the sensor may have been located in intracellular compartments [64]. It was not possible to identify neuron types from the images. Some labeled neurons were fairly large (up to 25 µm in diameter), and could therefore be pyramidal cells (e.g., Figure 4A–4C).

Recent findings suggest that the relative proportions of transfected excitatory and inhibitory neurons are strongly dependent on the virus titer of the AAV1 [53]. Thus, with further developments of viral vectors and membrane proteins, selected targeting of neuronal subtypes should become realistic. For example, a virally-based system that transfects anterograde or retrograde projections will be advantageous, as it reveals how individual connections can underlie neuronal selectivity [65].

### Optical Chamber

Scanning was performed in existing chamber implants with a transparent artificial dura that allowed unobstructed access to superficial cortex for months or years [30], [31], [32]. The thin silicone artificial dura used extensively for intrinsic optical imaging [66], [67] also allows repeated injections of virus material using sharpened micropipettes. Certain problems might arise with this preparation. Dural tissue can regrow over the cortex and underneath the artificial dura. Furthermore, buildup of cerebrospinal fluid occurred in M4R making it difficult to bring the Microprobe lens close enough to the cortical surface. Excessive fluid could be released by lifting the artificial dura immediately prior to recording.

### Microscope Lens

Regular high numerical aperture, water-immersion objectives were too wide to fit into the optical chamber. A narrow and elongated, water-immersion microscope objective (Olympus Microprobe, 27X, 0.7 NA) was the only lens that allowed access to the cortex within the chamber. Under visual guidance, the lens was easily aligned with the blood vessel pattern to locate the injection site. In many experiments, neurons were imaged at relatively high magnification, so that more details became apparent. For example, the sites displayed in Figure 3B and 3C show visible processes. Another site (e.g., Figure 4A–4C) was imaged repeatedly under high magnification showing the typical appearance. The cell membrane and cross sections of processes are distinctly and brightly labeled. Other experiments demonstrated that groups of neurons could be imaged at once at lower magnification (*Neurons 4–7*, Figure 9). Thus, functional scanning of single neurons is feasible both at high and low magnification.

Despite these advantages, the imaged neurons appeared blurred, and mostly the cell bodies were visible. For example, images of *Neuron 3* did not show much detail even at higher magnification. Decrement in optical quality of the imaged cells might be attributed to the angle of scanning (from the top of cortex surface), fluid between artificial dura and cortex surface, and tissue growth on top of brain or underneath the artificial dura. With improvement of the optics, specifically a higher numerical aperture objective, it should become feasible to image cell bodies *and* processes. Identification of the neural morphology to distinguish pyramidal neurons from other cell types should be possible. In addition, a longer working distance of the lens would enable recording from greater depths. The depth limits with TPSM are in the range of 600–800 µm depending on various factors such a wavelength, laser pulse shape and tissue properties [68].

### Rigidity and Movement

The TPSM recordings in the two monkeys M3R and M4R were extremely stable with little evidence of respiratory and cardiac induced movements. Thus, the rigid TPSM design is suitable for sub-micrometer imaging as shown previously in small rodents [9]. Furthermore, it should be emphasized that the FRET-based calcium sensor memTNXL is highly advantageous for studies in the behaving monkey. The ratiometric measurement decreases motion artifacts and correlated noise in the two channels. However, it is important to note that uncorrelated noise will increase if the denominator (CFP fluorescence) approaches zero [45].

Variability in FRET signal due to visual stimulation ranged from 0.02% to 5% ΔR/R. Many of the monkey FRET signals were smaller than those reported for cultured hippocampal neurons and transgenic *Drosophila* preparations (up to 100%, [39], [45]), and for a related sensor (TNXXL) in mouse visual cortex (up to 9%, [46]). However, it is difficult to relate the monkey FRET signals to these studies because of the differences in species, preparations, and methods of expression of the sensor.

### General Outlook and Improvements

Two-photon imaging of virally transformed neurons that express the fluorescent calcium sensor memTNXL in the behaving monkey permits the evaluation of morphology and neural activity. In two monkeys, neurons survived for many months after viral gene delivery and showed directional tuning. The current study is the first to successfully demonstrate TPSM of both structure and function at micrometer scale in the behaving monkey.

Nonetheless, there are crucial aspects that require improvement. Labeled neurons could be located easily at the injection sites with 18.8% of the recorded neurons showing significantly tuned responses. With the related sensor TNXXL used in mouse visual cortex, 22% of labeled neurons were visually responsive. Compared to studies with the calcium indicator dye Oregon Green BAPTA-1 acetoxymethyl ester (OGB), where about 60% of labeled cells in cat visual cortex showed orientation tuned responses [7], [8], the numbers obtained with memTNXL or TNXXL are relatively low. Thus, future improvements of the sensor and other aspects are necessary.

First, memTNXL expression might vary, resulting in different signal to background ratios. With baseline normalization, the response strength varied considerably (0.02% to 5%) between cells and experiments. Even in cells that showed overall strong responses with the onset of visual stimulation, tuning was often not significant. As shown in structural images, labeled neurons were clearly visible at different depths. To our knowledge there are no studies specifically comparing expression of these genetically encoded calcium sensors in primate cortex with other mammalian or non-mammalian species.

A second related question is whether the FRET signal of the current memTNXL sensor is strong enough in primate species when imaged under the conditions of the current study. The memTNXL sensor is designed to detect calcium influx through the plasma membrane during action potential activity. To our knowledge, this is the first attempt to use a membrane-targeted genetically encoded calcium sensor in the mammalian cortex *in vivo*. Recent improvement in the troponin C-based calcium sensors have yielded TNXXL [46]. In addition, there have also been considerable improvements of the single fluorophore GCaMP sensors, with the advent of G-CaMP2 and G-CaMP3 sensors [11], [40], [69], [70]. Membrane targeted versions of these new sensors may considerably improve the neural signal in the monkey preparation.

A third factor to consider is the complex relationship between the fluorescence change of a calcium sensor and neuronal firing rate *in vivo*. With genetically encoded calcium sensors the relationship between firing and fluorescence change is difficult to interpret [45]. A simple linear relationship might not apply to mammalian cortex, especially when using the method of viral transfection. Other related studies using troponin C-based calcium sensors (TN-L15 and TN-XXL) demonstrated a linear relationship between number of action potentials and increases in FRET ratio in cultured and slice hippocampal preparations [46], [71], [72]. Thus, more information is needed about these sensors and their “behavior” in multiple species using different methods for transgene expression [73]. Further complications might arise from neurons with low firing rates, in which fluorescence changes of genetically encoded calcium sensors might not be reliable. In primate visual cortex, in particular in superficial layers [60], [74], neurons often do not have very high firing rates and changes in fluorescence due to a small increase in spiking (e.g., 0–10 spikes/s) might not be detectable [75].

The fourth factor is unrelated to the sensor properties or the TPSM technology, but rather to specific neural properties in V1/V2, is suboptimal visual stimulation or insufficient number of trials per condition. The stimuli were always square wave gratings of fixed spatial frequency and movement speed. With 8–10 presentations per condition (i.e., grating direction), these stimuli might simply not be efficient enough to elicit a strong response in V1/V2 neurons. Certainly, averaging across more trials might be necessary to improve the signal to noise ratio. As scanning was performed in the superficial layers, a substantial number of labeled neurons might be very sensitive to stimulus length [76], [77], [78] and therefore responding weakly to these full field gratings. Thus, for future studies in V1/V2, stimuli would have to be optimized and include a wider variety of parameters.

In conclusion, we demonstrate successful TPSM achieved in the awake, behaving monkey performing a visual task. This methodology will serve a crucial role in understanding neuronal computations as proposed by Francis Crick at the end of the seventies [79]. Crick stressed that there were biological problems to clarify the brain's roles in interpreting and understanding single unit activity. For example, he proposed that it essential to know how many cells a neuron would be connected to. After thirty years this was answered [65]. These questions as to the connectivity of the brain are now being solved by the applications of molecular and systems neuroscience and ultimately will include the two-photon imaging methods described here. Crick (1979) had said that “... the new methods bring new results and new results foster new ideas, so we should not be too easily discouraged.” We believe that TPSM will be applied to the early cortical areas as well as complex areas such as the inferior parietal and inferior temporal areas. It is perhaps these most complex areas that will ultimately yield answers on how such cognitive functions arise from in single neurons and neuronal assemblies.
